# Prevalence of β-hemolytic *Streptococcus* in children with special health care needs

**DOI:** 10.5935/1808-8694.20120017

**Published:** 2015-11-20

**Authors:** Viviane Martha Santos de Morais, Alice Ramos Orsi, Fernanda Cristina de Albuquerque Maranhão, Therezita Maria Peixoto Patury Galvão Castro, Karina Cavalcante Beltrão de Castro, Denise Maria Wanderlei Silva

**Affiliations:** a Biologist, Federal University of Alagoas (MSc. student in the Department of Tropical Medicine, Federal University of Pernambuco); b Undergraduate student (undergraduate student, Biologic Sciences Program, Federal University of Alagoas); c PhD in Genetics, University of São Paulo (Adjunct Professor in the Microbiology Sector of the Institute of Biologic Sciences and Health, Federal University of Alagoas); d PhD in Medicine, Santa Casa de São Paulo (Adjunct Professor in the Medical School Surgical Practice Sector, Federal University of Alagoas); e PhD in Molecular Biology, University of Georgia (Associate Professor in the Microbiology Sector of the Institute of Biologic Sciences and Health, Federal University of Alagoas). Universidade Federal de Alagoas

**Keywords:** disabled children, down syndrome, Streptococcus, Streptococcus pyogenes

## Abstract

Pharyngotonsillitis by β-hemolytic *Streptococcus* mostly affects children and imunocompromissed, being *Streptococcus pyogenes* (Group A) the most common agent in bacterial pharyngotonsillitis.

**Aim:**

This work targeted the research of β-hemolytic *Streptococcus* Group-A (SBHGA) and No-A (SBHGNA) in the oropharynx of individuals with special health needs from the APAE (Maceió-AL).

**Method:**

A prospective study with oropharynx samples from patients with Down syndrome and other mental disorders (test) and students from a private school (control) aged 5-15 years. Cultures in blood agar (5%) were identified through Gram/catalase tests and bacitracin/trimethoprim-sulfamethoxazole disk diffusion method, applying the chi-squared statistical analysis.

**Results:**

A total of 222 bacterial colonies were isolated in 74 individuals from APAE and 65 in the control group. In the test group, previous episodes of pharyngotonsillitis were reported by 36.49% (27/74) and 9.46% (7/74) were diagnosed with symptoms and/or signs suggestive of oropharynx infection. No positive sample of *S. pyogenes* was confirmed at APAE, being all samples classified as SBHGNA, with 5 SBHGA in the control group.

**Conclusion:**

The early identification of β-hemolytic *Streptococcus* is important for the fast treatment of pharyngotonsillitis and the absence of *S. pyogenes* avoid future suppurative or not-suppurative sequels in the group from APAE.

## INTRODUCTION

Pharyngotonsillitis by *Streptococcus* affects more frequently pediatric patients aged ranging between three and 16 years^1^. Children frequenting day care centers may have incidence rates of respiratory tract infections up to 18 times greater than individuals of the same age who do not go to crowded enclosed spaces, and require antimicrobial drug therapy after complementary diagnosis^2^. However, individuals of all ages are susceptible to the propagation of this microorganism in conditions of overcrowding, as its interpersonal dissemination occurs through sputter or by direct contact, mainly in environments where large crowds get together, such as schools and day care centers^3^. Although bacterial infection accounts for only 10% of the cases of pharyngotonsillitis, this disease remains as a significant public health problem, as it may affect a large number of children and dissemination occurs easily among patients, students, and immunocompromised individuals in closed spaces^4^^,^^5^.

The main etiological agent of bacterial pharyngotonsillitis is group A *Streptococcus* (*Streptococcus pyogenes;* or GAS). It is related to a wide range of diseases, including impetigo, urinary tract infections, septic arthritis, peritonitis, and toxic shock syndrome. More specifically, this bacterium is generally associated with upper respiratory tract infections such as tonsillitis and pharyngitis, and also causes bronchitis. In addition to that, undiagnosed untreated episodes may lead to suppurative and non-suppurative sequelae such as glomerulonephritis and rheumatic fever respectively^6^^,^^7^.

The epidemiological observation of this pathogen is relevant due to the existence of complications and ease of transmission. Infections need to be managed more adequately and sequelae prevented by offering proper treatment to patients suspected for and diagnosed with infection^8^. Quick detection methods are of paramount importance in isolating β-hemolytic *Streptococcus*, both GAS and non-GAS in suspected cases, thus avoiding the injudicious use of antimicrobial drugs and the onset of sequelae, mainly in immunocompromised individuals such as subjects with genetic syndromes with associated comorbidities.

This study looked into the prevalence rates of β-hemolytic *Streptococcus* and the presence of *S. pyogenesin* the oropharynx of children and adolescents with special needs seen at APAE (Associação de Pais e Amigos dos Excepcionais) in Maceió, AL, Brazil, between October of 2008 and June of 2009. The control group was made up of chidren and adolescents from a private school located in Maceió-AL.

## METHOD

This study was approved by the Ethics Committee of our institution and given permit n° 018326/2008-08 and n° 010947/2008-35. The parents or guardians of the individuals enrolled in the study signed an informed consent term. This prospective cross-sectional historical cohort study included children and adolescents with Down syndrome and other mental disorders (congenital cerebral palsy and moderate to severe mental retardation with associated physical impairments) seen at APAE, Maceió-AL aged between 5 and 15 years in the case group. The control group included students from a private school located in the same region and within the same age range. The subjects from both groups had similar confinement routines in the institutions they attended and spent part of the day in a room with other children and adolescents. Individuals under five or over 15 years of age, subjects who were not fasting, who had done oral hygiene on sampling day, or who had been taking antibiotics recently were not enrolled in the study (exclusion criteria). General and clinical information was obtained through standard questionnaires. Subjects had their oral cavities examined before oral sampling to identify symptoms of infection such as throat inflammation, hypertrophied tonsils, purulent plaque, and other factors such as local pain, fever, nasal obstruction, coughing, and symptoms experienced previously.

Oropharyngeal secretion samples were taken from 74 children in the case group and 65 in the control group between October of 2008 and June of 2009. The protocol proposed by Winn et al.^9^ was used in order to standardize the lab workup used to identify the presence of GAS. Each swabwas transported in saline solution (0.9% NaCl) and each sample inoculated into a sheep blood agar slide (5%). After 24h-48h of candle jar incubation at 35°C, colonies suggestive of β-hemolytic *Streptococcus* were collected and transferred to a brain and heart infusion (BHI, Difco). Gram staining and biochemical tests were then performed to find whether there was catalase activity. The bacitracin susceptibility test using the disk diffusion method is presumptive for identification based on GAS (S. *pyogenes)* sensitivity to 0.04 U of bacitracin. This application was used together with the sulfamethoxazole-trimethopim susceptibility test in sheep blood agar (5%) with disks containing respectively 1.25 μg and 23.75 μg. This method can differentiate between groups of strep, once other groups of streptococci are sensitive and resistant to GAS. The samples collected in the control group were submitted to the same procedures described above, and a standard strain of *S. pyogenes* (ATCC 19615) was used in all experiments. The results were assessed through descriptive statistics, and the chi square test (χ^2^) was used to analyze the possible associations between symptomatic and asymptomatic subjects and children of different age ranges with and without previous involvement by pharyngotonsillitis. On all cases, *p* < 0.05 (5%) was used as a reference to reject the null hypothesis (H0).

## RESULTS

Thirty-six of the 74 subjects in the case group had Down syndrome (48.64%), while the remaining individuals had various other mental disorders. In this group, previous episodes of pharyngotonsillitis were reported by 36.49% (27/74) of the subjects and prevalence rates were higher among individuals aged 6. Additionally, 9.46% (7/74) of the children were diagnosed with symptoms or signs suggestive of oropharynx infection such as hypertrophied tonsils, redness, throat pain, and painful swallowing. None of them had aspiration pneumonia.

Two hundred and twenty-two samples from 74 case group subjects were analyzed, as colonies were isolated in triplicate for each clinical sample collected and cultured. All samples in the case group were categorized as belonging to the *Streptococcus genus*, as they had β-hemolytic colonies after culture in 5% sheep blood agar and were Gram-positive and catalase-negative. Although sensitivity to bacitracin was confirmed in isolated β-hemolytic colonies, unexpected results were seen and sensitivity to sulfamethoxazole-trimethopim was observed for all 222 tested colonies.

Despite the clinical diagnosis of pharyngotonsillitis assigned to 9.46% (7/74) of the subjects, the presence of GAS was not confirmed in oropharyngeal secretion samples of case group individuals with active symptoms. Thus, only β-hemolytic *Streptococcus* was detected in the obtained test material. Species of *Streptococcus* sensitive to bacitracin and sulfamethoxazole-trimethoprim (BA-SXT) are categorized β-hemolytic in strep groups C, F or G and, despite the similarities, they are categorized as non-group A β-hemolytic *Streptococcus* (non-GAS). Nonetheless, in the control group the prevalence rates of GAS and non-GAS were 7.7% (5/65) and 6.15% (4/65) respectively, with 60% of the asymptomatic carriers reporting previous episodes of pharyngotonsillitis. Table 1 shows the subjective characteristics and the statistical prevalence rates in children and adolescents with special needs, focusing on symptomatic individuals and subjects with previous episodes of pharyngotonsillitis.

**Table 1:** Characteristics of individuals with special needs and reported previous pharyngotonsillitis.

Age	Patients (n/%)	Symptoms (n/%)	Previous pharyngotonsillitis (n/%)
5	9/12.16	0/0	2/2.7
6	8/10.81	1/1.35	6/8.11
7	6/8.12	0/0	2/2.70
8	7/9.46	0/0	1/1.35
9	6/8.12	1/1.35	2/2.70
10	10/13.51	2/2.70	5/6.76
11	9/12.16	2/2.70	3/4.05
12	6/8.12	0/0	3/4.05
13	4/5.41	0/0	1/1.35
14	2/2.70	0/0	1/1.35
15	7/9.46	1/1.35	1/1.35

Statistical analysis of case group subjects aged between five and 10 years and individuals aged between 11 and 15 was performed initially to elicit the differences in the number of asymptomatic and symptomatic individuals. The null hypothesis (H0) stated that the number of asymptomatic and symptomatic subjects is not correlated to age, while the affirmative hypothesis (H1) stated that age is correlated to the number of symptomatic and asymptomatic individuals. Statistical calculation indicated that χ^2^ was equal to 0.0828, thus smaller than *p* < 0.05 and indicative that H0 is true with the result of asymptomatic and symptomatic subjects between 42/4 (5-10 years of age) and 25/3 (11-15 years of age). Another comparison between subjects used 18/28 (5-10 years of age) and 9/19 (11-15 years of age) with and without previous pharyngotonsillitis respectively, indicating as H0 the lack of a correlation with age range and as H1 the existence of a correlation with age, confirming H0 as χ^2^ was equal to 0.3667 (*p* < 0.05).

The search for *Streptococcus* and *S. pyogenes* in the oropharynx of children with special needs was particularly important, as only non-GAS was found. Early identification allows for quick treatment and prevention of future episodes of strep pharyngotonsillitis in general and more specifically cases caused by *S. pyogenes*, thus avoiding suppurative and non-suppurative sequelae.

## DISCUSSION

The gold standard for the diagnosis of pharyngitis and pharyngotonsillitis is the culture of oropharynx secretion in 5% sheep blood agar, with sensitivity greater than 90%^3^^,^^9^^,^^10^. Researchers have confirmed that susceptibility to bacitracin offers presumptive identification with accuracy above 95% for GAS and ranging between 83%- 97% for non-group A streptococci^5^^,^^11^^,^^12^. GAS (*S. pyogenes*) is the most common cause of bacterial pharyngitis, but other streptococci such as group C and G β-hemolytic strep^5^ may also cause acute pharyngitis. Sensitivity to bacitracin is of 10% among group C, F, and G *Streptococci*; the sulfamethoxazole-trimethoprim test is recommended to differentiate GAS and non-GAS, as the latter group is sensitive to SXT^4^. Therefore, when oropharynx secretion culture is positive for non-GAS, the usual clinical indication is antimicrobial therapy for *S. pyogenes.*

The presumptive identification of *S. pyogenes* in oropharynx secretion culture may be reliable when based on sensitivity to bacitracin or a positive test for pyrrolidonilarilamidase(PYR)^3^. There are various biochemical and microbiological tests available for GAS, but bacitracin sensitivity is the most widely used technique because it is highly sensitive and inexpensive, albeit not very specific^13^^,^^14^. Tests with blood agar, Gram staining, and bacitracin have been used in early screening for *S. pyogenes* followed by antigen detection tests^10^.

Sitkiewicz & Hryniewicz^15^ have found that similarly to GAS, groups B (GBS), C (GCS), and G (GGS) streptococci may be carried by humans and can cause diseases with symptoms identical to classic pharyngitis, but are rarely involved in invasive pyogenic infection. For five years in Buenos Aires, Argentina, Villar et al.^16^ have looked into samples of pharyngeal secretion of children aged between six months and 18 years and adults, and found non-GAS in 5.8% and 18.9% of the subjects in each respective group, to conclude that special care is to be given not only to *S. pyogenes*, but also to other streptococci as cases of invasive infection by non-GAS in immunocompromised individuals have been reported.

Molecular analysis is increasingly used to subtype the various groups of streptococci and assess their virulence. In a broad study done in India^17^, the authors sent 1,040 samples of patients with acute pharyngitis for molecular typing based on *emm* genotypes and superantigens. In the end they found 124 individuals with β-hemolytic strains, 59.7% with GCS, 25% with GGS, and 15.3% with GAS, stressing once again the epidemiological relevance of non-GAS in active infections, in this case verified through Multiplex PCR. In 2011, another molecular study on β-hemolytic GAS, GCS, and GGS isolated 63% of non-invasive strains typed as non-GAS^18^.

Subjects with Down syndrome account for the majority of the case group individuals enrolled in this study, and are more frequently affected by episodes of infection as a result of immune system anomalies and possibly clinical complications arising from the syndrome^19^. Case group individuals in this study were more susceptible to infection or colonization by non-GAS when compared to controls. Proper practice dictates that asymptomatic and symptomatic subjects with any bacterial infection be identified through adequate clinical examination and workup as required. Generation of more epidemiological data on the prevalence of pathogens in children with special needs is needed, as only a few studies have been carried out on this group of subjects in specialized institutions. Further research may improve the clinical care provided to these individuals.

## CONCLUSION

Non-group A β-hemolytic streptococci (non-GAS) were more frequently found in school-aged children with special needs when compared to control group subjects, thus eliciting their increased susceptibility. All subjects carried β-hemolytic streptococci regardless of having had previous episodes of pharyngotonsillitis. It has been confirmed that individuals with Down syndrome and immunocompromised subjects deserve special attention as they are more susceptible to infectious diseases.
